# Recent Work on the Nature and Development of Delusions

**DOI:** 10.1111/phc3.12249

**Published:** 2015-09-04

**Authors:** Lisa Bortolotti, Kengo Miyazono

**Affiliations:** ^1^ University of Birmingham; ^2^ Keio University

## Abstract

In this paper we review two debates in the current literature on clinical delusions. One debate is about what delusions are. If delusions are beliefs, why are they described as failing to play the causal roles that characterise beliefs, such as being responsive to evidence and guiding action? The other debate is about how delusions develop. What processes lead people to form delusions and maintain them in the face of challenges and counter‐evidence? Do the formation and maintenance of delusions require abnormal experience alone, or also reasoning biases or deficits? We hope to show that the focus on delusions has made a substantial contribution to the philosophy of the mind and continues to raise issues that are central to defining the concept of belief and gaining a better understanding of how people process information and learn about the world.

## 1 Introduction

In this paper we shall offer an overview of two lively debates in the philosophical and empirical literature on delusions. First, we focus on the debate about *what delusions are*. Then, we turn to the debate about *how delusions develop*. The two debates are interrelated: fundamental issues about the nature of delusions are reflected in the search for a theory about how delusions develop. Considerations about the nature and development of delusions start from a reflection on clinical cases in psychiatry and empirical work in cognitive neuroscience, and inform a broader theoretical understanding of how the mind works, what its limitations are and how perceptual, cognitive and affective states interact.

In the literature we review in this paper, the term ‘delusion’ refers to a clinical phenomenon, and in particular to an observable symptom of schizophrenia, delusional disorders, dementia, amnesia and other psychiatric disorders. In the most recent version of the *Diagnostic and Statistical Manual of Mental Disorders* (DSM‐5), delusion is defined as follows:

A false belief based on incorrect inference about external reality that is firmly held despite what almost everyone else believes and despite what constitutes incontrovertible and obvious proof or evidence to the contrary. The belief is not ordinarily accepted by other members of the person's culture or subculture (i.e., it is not an article of religious faith). When a false belief involves a value judgment, it is regarded as a delusion only when the judgment is so extreme as to defy credibility (American Psychiatric Association).

The definition has been challenged on various grounds (Coltheart), but it remains a useful diagnostic tool for clinicians.

Examples of delusions are *persecution*, where the person reports that other people are hostile and intend to cause her harm, and *jealousy*, where the person reports that her romantic partner is being unfaithful to her. More bizarre delusions include *mirrored‐self misidentification*, where the person reports that there is a stranger in the mirror, or the *Cotard delusion*, where the person reports that she is dead or disembodied. Despite differences in content, delusions have similar characteristics, and they all tend to be maintained in the face of counter‐evidence and counter‐argument. We shall consider some features of delusions in more detail in the next section.

## 2 The Nature of Delusions

In this section, we shall review two approaches to the nature of delusions. The first approach is to regard delusions as beliefs (*doxasticism*). Doxasticists agree that delusions are beliefs (Bortolotti; Bayne and Pacherie; Miyazono and Bortolotti; Reimer), but they may disagree on whether delusions are *rational* or *irrational* beliefs, and about whether they are *continuous with* or *categorically different from* non‐delusional beliefs.

The second approach is to regard delusions as something other than beliefs (*anti‐doxasticism*). Anti‐doxasticists agree that characterising delusions as beliefs is unsatisfactory, but they disagree on the positive account that best captures the nature of delusions. Although some argue that delusions are empty utterances with no meaning (Berrios), most anti‐doxasticists defend the view that delusions are mental states of a non‐doxastic or a non‐exclusively‐doxastic nature: acts of imagination (Currie and Ravenscroft; Currie and Jureidini), perceptual inferences (Hohwy and Rajan), states in‐between beliefs and non‐beliefs (Schwitzgebel; Tumulty), states in‐between beliefs and desires or in‐between beliefs and imaginings (Egan), attitudes towards mental states (Stephens and Graham), acceptances (Frankish) or thoughts expressing the content of default processes (Gerrans).

Here we shall present some arguments for and against the claim that delusions are best understood as beliefs. In particular, we shall argue that, if we adopt a psychologically realistic view of the relationship between beliefs and evidence, and acknowledge the role that affective and motivational states play in driving action, anti‐doxasticism can be resisted.

### 2.1 Responsiveness to Evidence

Here is a common argument against the doxastic nature of delusions, the ‘argument from responsiveness to evidence’:

R1 It is a constitutive feature of beliefs that they are responsive to evidence.
R2 Delusions are not responsive to evidence.
R3 Therefore, delusions are not beliefs.

To evaluate this argument we need to consider the plausibility of (R1) and (R2).

Let us start with (R2). Are delusions responsive to evidence? Some definitions of delusions point to resistance to counter‐evidence as the main feature of delusions (Gilleen and David). This feature is also an important diagnostic criterion for delusions. According to DSM‐5, delusions are ‘fixed beliefs’. It would be unwise to claim that *all* delusions are *equally* impervious to revision, but empirical evidence speaks in favour of the view that most delusions are not responsive to counter‐evidence. For instance, in one striking case of the Cotard delusion (the delusion that one is dead or disembodied), a young woman continued to hold that she was dead, even after she acknowledged that dead people do not usually move and talk, and that she did move and talk (McKay and Cipolotti). In another powerful case report, a woman with erotomania (the delusion that one is loved by another, usually a stranger) continued to believe that a man was in love with her and intended to propose to her, even after she talked to him on the phone and he told her that he was not in love with her and could barely remember who she was (Jordan and Howe). This does not mean that delusional beliefs are completely insensitive to evidence though. People tend to argue for the content of their delusions when they encounter challenges. Moreover, the conviction in the delusions may fluctuate, and sometimes it does so as a result of experience, reflection or external challenges.

Let us consider (R1) now. Do beliefs need to be responsive to evidence? There are several philosophical and psychological accounts of what constitutes a belief, but there is very little agreement on the necessary and sufficient conditions for belief. Thus it is hard to argue for or against the doxastic nature of delusions on the basis of what we take beliefs to be. Many accounts converge on the fact that responsiveness to evidence is a key feature of beliefs, a feature that, for instance, distinguishes beliefs from acts of imagination or wishful thinking. But it is debatable whether responsiveness to evidence is a criterion for belief or, rather, an ideal to which rational beliefs aspire. We seem to categorise some attitudes as beliefs even if they are not responsive to evidence. In some circumstances, we form beliefs, and uphold them, independently of whether there is evidence supporting them. In other circumstances, we form beliefs based on evidence, but we do not easily update them or reject them when counter‐evidence emerges. Examples include prejudiced, superstitious and religious beliefs (Brewster and Rusche), beliefs in scientific theories we are committed to (Chinn and Brewer) and beliefs about ourselves in general, including beliefs about our traits, talents, weaknesses and ideological convictions (Markus; Pyszczynski, Greenberg, and Holt).

Thus, the argument from responsiveness to evidence does not seem to be conclusive as an argument against the doxastic nature of delusions. Doxasticists may respond to the argument by acknowledging that delusions tend to be resistant to counter‐evidence, but pointing out that other instances of belief do so too. This puts pressure on anti‐doxasticists to either provide additional reasons to doubt the doxastic nature of delusion or conclude that other attitudes commonly described as beliefs are not genuine instances of beliefs because of their resistance to counter‐evidence. Anti‐doxasticists can respond to this challenge, but here we shall not examine the debate further.

### 2.2 Action Guidance

Another common argument against doxasticism is the ‘argument from action guidance’:

G1 It is a constitutive feature of beliefs that they guide action.
G2 Delusions do not guide action.
G3 Therefore, delusions are not beliefs.

To evaluate this argument we need to consider the plausibility of (G1) and (G2).

Let us start with (G2). Do delusions really fail to guide action? It is a diagnostic criterion for delusions that they lead to action, and thus it is implausible to claim that delusions *generally* fail to guide action. People have been known to engage in risky behaviours, stop contacting their families, move cities, abandon their studies and change jobs as a result of their delusions. But some philosophers have commented on the phenomenon of *double bookkeeping* that seems to apply to at least some delusions. Double bookkeeping refers to the presence of conflicting commitments within an agent and is usually exemplified by people who make verbal reports that are not reflected in their behaviour. For instance, in two classic examples, a woman claims to be the queen but does not behave like royalty (Bleuler), and a man claims that the nurses in the hospital want to poison him but eats the food they give him (Gallagher). In such circumstances it is legitimate to ask whether the woman genuinely believes she is the queen, and whether the man is serious in his allegations against the hospital nurses.

What about (G1)? Action guiding is an important feature of beliefs, and, just like responsiveness to evidence, it may help us distinguish beliefs from other attitudes. Believing that an untamed lion is in the room may lead us to run away, whilst imagining a lion in the room may not have the same effect. That said, as with responsiveness to evidence, not all beliefs share the same action‐guiding potential. Some beliefs have a direct impact on behaviour and some do not. Some beliefs are acted on in some circumstances and not in others. The psychological literature on everyday hypocrisy shows that cases of double bookkeeping are by no means confined to delusions.

One important consideration is the role played by motivation in turning beliefs into actions. Even the most strongly held belief may not be acted on if independent factors inhibit motivation, such as the agent lacking confidence, fearing a sanction or missing the opportunity to act. In the psychiatric disorders characterised by delusions, and especially in schizophrenia, motivation is negatively affected. This might mean that genuine beliefs can generate the appropriate intentions, but fail to play their action‐guiding role if such intentions are not carried out and converted into action. This phenomenon provides some explanation for the apparent lack of commitment to some delusional states observed in the literature on double bookkeeping (Bortolotti and Broome).

### 2.3 Revisionism or Conservatism About Belief

The arguments discussed in the previous sections may not be convincing on their own, but they have been powerfully combined in the work of some influential anti‐doxasticists. If delusions fail to be responsive to evidence and they also fail to guide action in the relevant circumstances, one may deny that they have the same causal roles as beliefs and wonder whether it is helpful to regard them as beliefs at all.

In light of this discussion, two options emerge. *Revisionism* recommends that we should change our terminology. We should call ‘beliefs’ only those attitudes that are responsive to evidence and guide action (i.e. that play the causal roles of beliefs), and find some other term to refer to those attitudes that share many of the features of beliefs, but are less responsive to evidence and less influential on action than typical beliefs. Authors who pursue this strategy in different ways include Greg Currie, Keith Frankish, Dominic Murphy, Eric Schwitzgebel and Andy Egan. The worry with this particular revisionist strategy is that it does not necessarily lead to a philosophy of belief that is more compatible with the psychological science or our everyday experience of believers. Instead, it is an attempt to preserve an idealised notion of belief that applies only to attitudes conforming to norms of rationality for beliefs. If followed through, this move would make the category of belief much less populated.

*Conservatism* recommends that we should not change our terminology and that we continue to use the term ‘belief’ quite liberally. The category of belief needs to be demarcated somehow, and it cannot be stretched to encompass those attitudes entirely lacking impact on behaviour or sensitivity to evidence. But it should not be required of all attitudes to be responsive to evidence and to be action‐guiding to count as beliefs. Those who are conservative about the notion of belief have no reason to doubt the doxastic nature of delusions. Just like other attitudes that can be resistant to evidence or that fail to guide action, delusional states can count as beliefs. After all, ascribing delusions often serves the same purpose as ascribing beliefs: it allows agents to predict and understand other agents' behaviour, coordinate and cooperate.

Revisionism and conservatism can be both plausibly defended, and the budding literature on the topic shows that philosophers can make sense of many features of delusions independently of what strategy they opt for. One thing that cannot be ignored is that, whether we regard delusions as beliefs or as something else, many of their epistemic features are shared by attitudes that are widespread in the non‐clinical population, suggesting continuity between so‐called normal and abnormal cognition.

## 3 The Development of Delusions

In this section, we shall review two approaches to the development of delusions (i.e. the formation and maintenance of delusions) that are popular in the empirical literature: two‐factor accounts and prediction‐error accounts. We shall suggest that, despite being usually presented as incompatible, the two approaches can be integrated.

### 3.1 Two‐factor Theories

Two‐factor theories (Coltheart; Coltheart, Langdon, and McKay; Davies et al.) are very influential, especially as accounts of monothematic delusions (i.e. delusions concerning a single theme). The Capgras delusion is the most extensively discussed example. In the Capgras delusion the person comes to believe that a loved one has been replaced by an identical, or almost identical, impostor. Ellis and Young argue that the Capgras delusion is formed in response to an abnormal perceptual‐affective experience of seeing familiar faces. It is known that normal individuals exhibit stronger autonomic responses when perceiving a familiar face than when perceiving an unfamiliar face. However, the asymmetry in autonomic responses is missing in people with the Capgras delusion (Ellis et al.). When seeing familiar faces and having the abnormal experience people ‘adopt some sort of rationalisation strategy in which the individual before them is deemed to be an imposter, a dummy, a robot, or whatever extant technology may suggest’ (Ellis and Young 244).

Is the abnormal perceptual‐affective experience causally sufficient for the development of the Capgras delusion? Someone who is sympathetic to Maher's famous claim that ‘a delusion is a hypothesis designed to explain unusual perceptual phenomena and developed through the operation of normal cognitive processes’ (Maher 103) might think that it is causally sufficient. Two‐factor theorists, however, do not think that the abnormal experience is sufficient for the development of the Capgras delusion. In addition to the abnormal experience (the first factor) there has to be an additional factor. The main argument for the second factor goes as follows: just like people with the Capgras delusion, people with damage to the ventromedial prefrontal cortex (vmPFC) fail to show the asymmetrical autonomic responses between familiar and unfamiliar faces (Tranel, Damasio, and Damasio), which suggests that people with the Capgras delusion and with vmPFC damage have a similar abnormal experience. However, people with vmPFC damage do not develop the Capgras delusion. The best explanation of this is that the abnormal experience is not sufficient for the development of the Capgras delusion, and the second factor explains why people with vmPFC damage do not necessarily develop the Capgras delusion. Similar arguments about other monothematic delusions have been presented (Coltheart; Davies et al.). Thus, one fundamental commitment of two‐factor theories is that there are two factors contributing to the development of delusions. The two factors are dissociable from each other (i.e. one can exist without the other), and the first factor is not causally sufficient for the development of delusions.

There is no consensus about the nature of the two factors, but it is widely assumed that the two factors play different explanatory roles. In particular, the first factor explains the *content* of the delusion, whilst the second factor explains the *development* of the delusion. More precisely, the second factor explains either why the delusion is formed and maintained (e.g. Stone and Young; McKay) or only why the delusion is maintained (e.g. Coltheart, Menzies, and Sutton).

The main disagreement among different versions of the two‐factor theory concerns the nature of the second factor. Stone and Young as well as McKay argue that the inferential step from the first factor (e.g. an abnormal perceptual‐affective experience of seeing familiar faces) to the delusional belief (e.g. ‘this woman is not my wife’) involves biased reasoning. According to their version of the two‐factor theory, the reasoning bias is the second factor. Coltheart, Menzies, and Sutton, on the other hand, claim that the inferential step from the first factor to the delusional belief is perfectly rational from a Bayesian point of view. According to their version of the two‐factor theory, the second factor is not a reasoning bias but rather the unreasonable maintenance of delusions despite overwhelming counter‐evidence.

### 3.2 Prediction‐error Theories

Prediction‐error theories (Adams et al.; Clark; Corlett, Murray, et al.; Corlett, Taylor, et al.; Fletcher and Frith; Hohwy) are emerging theories that are primarily aimed at explaining delusions in schizophrenia. The fundamental idea of prediction‐error theories is that delusions are the product of the abnormalities in the belief‐updating processes driven by prediction errors.

A prediction error is, in general, the mismatch between an expectation and an actual input. A remarkable study (Corlett, Murray, et al.) suggests that prediction‐error signalling is abnormal in people with delusions in the context of schizophrenia. In the study, two groups of subjects, people with delusions due to schizophrenia and non‐clinical controls, were tested in a task involving learning and predicting the association between certain foods and allergic reactions to them. The activity of the right prefrontal cortex (rPFC) (that had been identified as a reliable marker of prediction‐error processing in previous studies) was monitored with fMRI. Results showed that, in the control group, the rPFC activity was much greater in the cases where their predictions were disconfirmed than in the cases where they were confirmed, as if the brain was ‘surprised’ when the predictions were disconfirmed but not when they were confirmed. In the delusional group, the asymmetry in the rPFC activity was diminished, as if the brain was ‘surprised’ not only when the predictions were disconfirmed but also when they were confirmed.

The prediction‐error theory relies on the predictive‐processing framework for understanding brain and mind (for reviews, see Clark; Friston; Hohwy). The framework has the following core assumptions. First, the brain tries to minimise prediction errors, often (but not always) by updating expectations in a Bayesian manner on the basis of prior expectations and actual inputs. Second, prediction‐error minimisation processes occur in many different but interrelated levels of the processing hierarchy within the brain. Different levels deal with information at different degrees of abstractness (e.g. sentences, words, letters and shapes). A prediction error at the lower level becomes an input for the higher level, and, at the same time, feedback from the higher level becomes an expectation at the lower level. Third, not all prediction errors are processed in the same way. The brain is sensitive to *precision*, that is, the indicator of the reliability or trustworthiness of prediction errors. Precise prediction errors are weighted more strongly in the hierarchical processing than imprecise prediction errors.

In this framework, belief updating is just a process of prediction‐error minimising at some levels (presumably, some higher levels) of the hierarchy. A consequence of this view is that delusions might be conceptualised as the product of abnormalities in the belief‐updating processes that are driven by prediction errors. But what is the nature of these abnormalities? A popular view is that they involve the failure to estimate the precision of sensory prediction errors. For instance, some sensory prediction errors that would usually be ignored are regarded as extremely precise, and thus they propagate upward in the hierarchy causing a revision of prior beliefs. This seems to fit nicely with what we know about the way in which delusions in schizophrenia are typically formed. In the prodromal phase of schizophrenia (i.e. the early stage before the onset of specific symptoms), random events that would be usually ignored become *salient* (Kapur). They suddenly grab attention, acquire importance and demand an explanation. For instance, one may be drawn to the pattern of the colours of the doors on a street and wonder if there is a reason for the pattern. The delusional belief, say, that the pattern conveys a secret message from an unknown organisation, is accepted as the explanation of the abnormally salient event.

The main disagreement among different versions of the prediction‐error theory concerns the nature of the abnormality in the belief‐updating processes driven by prediction errors. The abnormality might involve the bottom‐up revision of prior (non‐delusional) beliefs due to the presence of highly precise sensory prediction errors (Adams et al.; Fletcher and Frith; Frith and Friston). This version of the prediction‐error theory nicely explains the process by which delusions are formed. Alternatively, the abnormality might involve the heavy reliance on top‐down (delusional) prior beliefs due to imprecise sensory prediction errors (Hohwy). This version of the prediction‐error theory nicely explains the process by which delusions are maintained despite overwhelming counter‐evidence.

### 3.3 How Do the Two Approaches Relate to Each Other?

Each of the approaches we described, the two‐factor theory and the prediction‐error theory, has some unresolved issues. As we noted, an unresolved issue for two‐factor theorists is the elusive nature of the second factor. An unresolved issue for prediction‐error theorists is the nature of the abnormality in prediction‐error processing.

In addition to those issues that are internal to each approach, there are unresolved issues concerning the relation between the two approaches. It seems as though both two‐factor theories and prediction‐error theories have a significant degree of theoretical and empirical plausibility. But how do they relate to one another? One view is that the two approaches do not share target phenomena in the first place. For example, one could argue that two‐factor theories explain the development of monothematic delusions, and prediction‐error theories explain the development of delusions in schizophrenia. This view, however, is not fully satisfactory because ‘monothematic delusions’ and ‘delusions in schizophrenia’ are not exclusive categories and can overlap. For instance, the Capgras delusion is a typical monothematic delusion, but it can also be found in the context of schizophrenia.

Another view is that the two approaches are incompatible and at least one of them is false. Prediction‐error theorists tend to hold this incompatibilist view. For instance, Corlett and colleagues argue that, if their account is correct, then there is no need for two distinct factors because

a single deficit in Bayesian inference is able to explain more of what we know about the interactions between perception and belief‐based expectation, the neurobiology of the delusions that occur in schizophrenia and the maintenance of delusions in the face of contradictory evidence (Corlett, Taylor, et al. 357).

But it is far from obvious that they can provide a plausible account of the asymmetry between people with the Capgras delusion and people with vmPFC damage without positing two separate factors.

A third view is that the two approaches are compatible and are correct (or, at least, some core ideas of both approaches are correct). This view seems to be more attractive than the competing views since it allows for the possibility of a hybrid account. The hope is that the hybrid account can inherit the theoretical and empirical plausibility of the two approaches and overcome the problems that the individual approaches still seem to have.

There are two possible compatibilist scenarios (Miyazono, Bortolotti, and Broome). According to one compatibilist scenario that could be articulated within a two‐factor theory approach, the first factor explaining the content of the delusion is related to prediction errors. For example, one might think that the first factor of the Capgras delusion is the mismatch between expected and actual affective responses to familiar faces (Coltheart, Langdon, and McKay). According to another compatibilist scenario that could also be pursued within a two‐factor theory approach, the second factor responsible for the development of the delusion is explained by prediction errors. For example, the inappropriately high precision of sensory prediction errors might be responsible for the bias that leads people with delusions to prioritise new observations over existing beliefs (McKay; Frith and Friston).

## 4 Concluding Remarks

The recent debates on the nature, formation and maintenance of delusions reflect interesting developments in the philosophy of mind broadly conceived. One question is whether we should hold on to our current folk‐psychological vocabulary or attempt to replace it with a new vocabulary driven by more sophisticated epistemological distinctions among the causal roles of various attitudes that we currently identify as beliefs. Another question is whether we should hold on to the distinction between experience and reasoning when we think about how we represent the world, or rather conceive of our minds and brains as prediction‐error minimising machines that process information at different levels of abstraction to conjure an overall model of the world that is constantly updated. Here we sketched a map of possibilities and suggested some promising ways forward.

## References

[phc312249-bib-0001] Adams, Rick A. , et al. ‘The Computational Anatomy of Psychosis.’ Frontiers in Psychiatry 4 47 (2013). 10.3389/fpsyt.2013.00047 PMC3667557 23750138

[phc312249-bib-0002] American Psychiatric Association . Diagnostic and Statistical Manual of Mental Disorders, Fifth Edition. 5th ed Washington, D.C.: American Psychiatric Publishing, 2013. Print.

[phc312249-bib-0003] Bayne, Tim and Elisabeth Pacherie . ‘In Defence of the Doxastic Conception of Delusions.’ Mind & Language 20 2 (2005): 163–88.

[phc312249-bib-0004] Berrios, G. ‘Delusions as ‘Wrong Beliefs’: A Conceptual History.’ British Journal of Psychiatry 159 (1991): 6–13. 1840782

[phc312249-bib-0005] Bleuler, E. Dementia Praecox or the Group of Schizophrenias. New York: International Universities Press, 1968.

[phc312249-bib-0006] Bortolotti, Lisa and Matthew R. Broome . ‘Affective Dimensions of the Phenomenon of Double Bookkeeping in Delusions.’ Emotion Review 4 2 (2012): 187–91.

[phc312249-bib-0007] Bortolotti, Lisa . Delusions and Other Irrational Beliefs. Oxford: Oxford University Press, 2009.

[phc312249-bib-0008] Brewster, Zachary W. and Sarah Nell Rusche . ‘Quantitative Evidence of the Continuing Significance of Race Tableside Racism in Full‐service Restaurants.’ Journal of Black Studies 43 4 (2012): 359–84. 22834051 10.1177/0021934711433310

[phc312249-bib-0009] Chinn, C. A. and W. F. Brewer . ‘The Role of Anomalous Data in Knowledge Acquisition: A Theoretical Framework and Implications for Science Instruction.’ Review of Educational Research 63 1 (1993): 1–49.

[phc312249-bib-0010] Clark, Andy . ‘Whatever Next: Predictive Brains, Situated Agents, and the Future of Cognitive Science.’ Behavioral and Brain Sciences 36 3 (2013): 181–204. 23663408 10.1017/S0140525X12000477

[phc312249-bib-0011] Coltheart, Max , Peter Menzies , and John Sutton . ‘Abductive Inference and Delusional Belief.’ Cognitive Neuropsychiatry 15.1/2/3 (2010): 261–87. 20017038 10.1080/13546800903439120

[phc312249-bib-0012] Coltheart, Max , Robyn Langdon , and Ryan McKay . ‘Delusional Belief.’ Annual Review of Psychology 62 (2011): 271–98. 10.1146/annurev.psych.121208.131622 20731601

[phc312249-bib-0013] Coltheart, Max . ‘The 33rd Sir Frederick Barlett Lecture: Cognitive Neuropsychiatry and Delusional Belief.’ The Quarterly Journal of Experimental Psychology 60 8 (2007a): 1041–62. 17654390 10.1080/17470210701338071

[phc312249-bib-0014] Coltheart, Max . ‘Cognitive Neuropsychiatry and Delusional Belief.’ Quarterly Journal of Experimental Psychology (2006) 60 8 (2007b): 1041–62. 10.1080/17470210701338071 17654390

[phc312249-bib-0015] Corlett, Phil R. , et al. ‘Disrupted Prediction‐error Signal in Psychosis: Evidence for an Associative Account of Delusions.’ Brain 130 (2007): 2387–400. 17690132 10.1093/brain/awm173 PMC3838942

[phc312249-bib-0016] Corlett, Phil R. , et al. ‘Toward a Neurobiology of Delusions.’ Progress in Neurobiology 92 (2010): 345–69. 20558235 10.1016/j.pneurobio.2010.06.007 PMC3676875

[phc312249-bib-0017] Currie, Gregory and Ian Ravenscroft . Recreative Minds. Oxford: Clarendon Press, 2002.

[phc312249-bib-0018] Currie, Gregory and Jon Jureidini . ‘Delusion, Rationality, Empathy: Commentary on Martin Davies et al.:’ Philosophy, Psychiatry, & Psychology 8 2 (2001): 159–62.

[phc312249-bib-0019] Davies, Martin , et al. ‘Monothematic Delusions: Toward a Two‐factor Account.’ Philosophy, Psychiatry, & Psychology 8 2/3 (2001): 133–58.

[phc312249-bib-0020] Egan, Andy . ‘Imagination, Delusion and Self‐deception’ Delusion and Self‐deception: Affective and Motivational Influences on Belief Formation. Eds. Bayne Tim and Fernández Jordi Hove, East Sussex: Psychology Press, 2009 263–80.

[phc312249-bib-0021] Ellis, Hadyn D. and Andrew W. Young . ‘Accounting for Delusional Misidentifications.’ British Journal of Psychiatry 157 (1990): 239–48. 2224375 10.1192/bjp.157.2.239

[phc312249-bib-0022] Ellis, Hadyn D. , et al. ‘Reduced Autonomic Responses to Faces in Capgras Delusion.’ Proceedings of the Royal Society Biological Sciences 264 (1997): 1085–92. 9263474 10.1098/rspb.1997.0150 PMC1688551

[phc312249-bib-0023] Fletcher, Paul C. and Chris D. Frith . ‘Perceiving is Believing: A Bayesian Approach to Explaining the Positive Symptoms of Schizophrenia.’ Nature Reviews Neuroscience 10 (2009): 48–58. 19050712 10.1038/nrn2536

[phc312249-bib-0024] Frankish, Keith . ‘Delusions: A Two‐level Framework’ Psychiatry as Cognitive Neuroscience: Philosophical Perspectives. Eds. Broome Matthew and Bortolotti Lisa Oxford: Oxford University Press, 2009 269–84.

[phc312249-bib-0025] Friston, Karl J. ‘The Free‐energy Principle: A Unified Brain Theory?’ Nature Reviews Neuroscience 11 2 (2010): 127–38. 20068583 10.1038/nrn2787

[phc312249-bib-0026] Frith, Chris D. and Karl J. Friston . ‘False Perceptions and False Beliefs: Understanding Schizophrenia’ Working Group on Neurosciences and the Human Person: New Perspectives on Human Activities. The Pontifical Academy of Sciences. Casina Pio IV, Vatican City. 8‐10 Nov. 2012.

[phc312249-bib-0027] Gallagher, Shaun . ‘Delusional Realities’ Psychiatry as Cognitive Neuroscience: Philosophical Perspectives. Eds. Broome M. R. and Bortolotti L. Oxford: Oxford University Press, 2009 245–66.

[phc312249-bib-0028] Gerrans, Philip . The Measure of Madness: Philosophy of Mind, Cognitive Neuroscience, and Delusional Thought. Harvard: MIT Press, 2014.

[phc312249-bib-0029] Gilleen, James and Anthony S. David . ‘The Cognitive Neuropsychiatry of Delusions: From Psychopathology to Neuropsychology and Back Again.’ Psychological Medicine 35 01 (2005): 5–12. *Cambridge Journals Online*. 15842024 10.1017/s0033291704003976

[phc312249-bib-0030] Hohwy, Jacob . The Predictive Mind. Oxford: Oxford University Press, 2013.

[phc312249-bib-0031] Hohwy, Jakob and Vivek Rajan . ‘Delusions as Forensically Disturbing Perceptual Inferences.’ Neuroethics 5 1 (2012): 5–11.

[phc312249-bib-0032] Jordan, Harold W. and Gray Howe . ‘De Clerambault Syndrome (Erotomania): A Review and Case Presentation.’ Journal of the National Medical Association 72 10 (1980): 979. 6999163 PMC2552541

[phc312249-bib-0033] Kapur, Shitij . ‘Psychosis as a State of Aberrant Salience: A Framework Linking Biology, Phenomenology, and Pharmacology in Schizophrenia.’ The American Journal of Psychiatry 160 1 (2003): 13–23. 12505794 10.1176/appi.ajp.160.1.13

[phc312249-bib-0034] Maher, Brendan A. ‘Delusional Thinking and Perceptual Disorder.’ Journal of Individual Psychology 30 1 (1974): 98–113. 4857199

[phc312249-bib-0035] Markus, Gregory B. ‘Stability and Change in Political Attitudes: Observed, Recalled, and ‘Explained’.’ Political Behavior 8 1 (1986): 21–44.

[phc312249-bib-0036] McKay, Ryan . ‘Delusional Inference.’ Mind & Language 27 3 (2012): 330–55.

[phc312249-bib-0046] Mckay, Ryan , and Cipolotti Lisa . ‘Attributional styles in a case of Cotard delusion.’ Consciousness and Cognition 16 (2007): 349–59. 16854594 10.1016/j.concog.2006.06.001

[phc312249-bib-0037] Miyazono, Kengo and Lisa Bortolotti . ‘The Causal Role Argument Against Doxasticism About Delusions.’ Avant V 3 (2014): 30–50.

[phc312249-bib-0038] Miyazono, Kengo , Lisa Bortolotti , and Matthew R. Broome . ‘Prediction‐error and Two‐factor Theories of Delusion Formation: Competitors or Allies?’ Aberrant Beliefs and Reasoning. Ed. Galbraith Niall Hove: Psychology Press, 2015 34–54.

[phc312249-bib-0039] Pyszczynski, Tom , Jeff Greenberg , and Kathleen Holt . ‘Maintaining Consistency Between Self‐serving Beliefs and Available Data: A Bias in Information Evaluation.’ Personality and Social Psychology Bulletin 11 2 (1985): 179–90.

[phc312249-bib-0040] Reimer, Marga . ‘A Davidsonian Perspective on Psychiatric Delusions.’ Philosophical Psychology 24 5 (2011): 659–77.

[phc312249-bib-0041] Schwitzgebel, Eric . ‘Mad Belief?’ Neuroethics 5 1 (2012): 13–7.

[phc312249-bib-0042] Stephens, G. Lynn and George Graham . ‘The Delusional Stance’ Reconceiving Schizophrenia. Eds. Cheung Chung Man, Fulford K. W. M., and Graham George Oxford: Oxford University Press, 2007.

[phc312249-bib-0043] Stone, Tony and Andrew W. Young . ‘Delusions and Brain Injury: The Philosophy and Psychology of Belief.’ Mind & Language 12 3/4 (1997): 327–64.

[phc312249-bib-0044] Tranel, Daniel , Hanna Damasio , and Antonio Damasio . ‘Double Dissociation Between Overt and Covert Face Recognition.’ Journal of Cognitive Neuroscience 7 4 (1995): 425–32. 23961902 10.1162/jocn.1995.7.4.425

[phc312249-bib-0045] Tumulty, Maura . ‘Delusions and Not‐quite‐beliefs.’ Neuroethics 5 1 (2012): 29–37. Print.

